# Resistance by applied immunology: fabricated typhus epidemic as civil protection in occupied Poland during World War II

**DOI:** 10.1007/s15010-025-02706-6

**Published:** 2026-02-06

**Authors:** Justyna Nunes-Biesiadecka, Dominika Drzewiecka, Sören Gatermann, Jonathan Jantsch, Gerd Fätkenheuer

**Affiliations:** 1 Division of Infectious Diseases, Department I of Internal Medicine, Faculty of Medicine and University Hospital of Cologne, University of Cologne, Cologne, Germany; 2 Department of Biology of Bacteria, Faculty of Biology and Environmental Protection, Institute of Microbiology, Biotechnology and Immunology, University of Lodz, Lodz, Poland; 3 Department of Medical Microbiology, University of Bochum, Bochum, Germany; 4 Faculty of Medicine and University Hospital of Cologne, Institute of Medical Microbiology, Immunology and Hygiene, University of Cologne, Cologne, Germany; 5 Center for Infectious Diseases, University Hospital of Cologne, Cologne, Germany

**Keywords:** Epidemic typhus, *Rickettsia prowazekii*, *Proteus vulgaris*, Weil–Felix test, Cross reactivity, O antigen

## Abstract

During World War II, Polish physicians Eugeniusz Łazowski and Stanisław Matulewicz fabricated a typhus epidemic, which they reported in detail only after the war. They injected inactivated *Proteus* bacteria to patients suffering from mild, flu-like ailments in order to trigger a positive Weil–Felix reaction in their serum, the standard diagnostic tool for typhus at that time. These falsely labelled typhus patients would then be protected from seizure by German occupiers, who were much concerned about transmission of this highly deadly disease. According to Łazowski and Matulewicz, this action saved many Poles from forced labour and other atrocities. We here show that this false epidemic was possible with the simple means available at that time, and that it is plausible from a medical and a historical perspective. How the two doctors combined medical textbook knowledge, social responsibility, epidemiological know-how and ingenuity under war conditions is outstanding. They should serve as role models for humanity and resistance under oppressive systems for present and future generations of physicians.

## Introduction

**Fig. 1:**
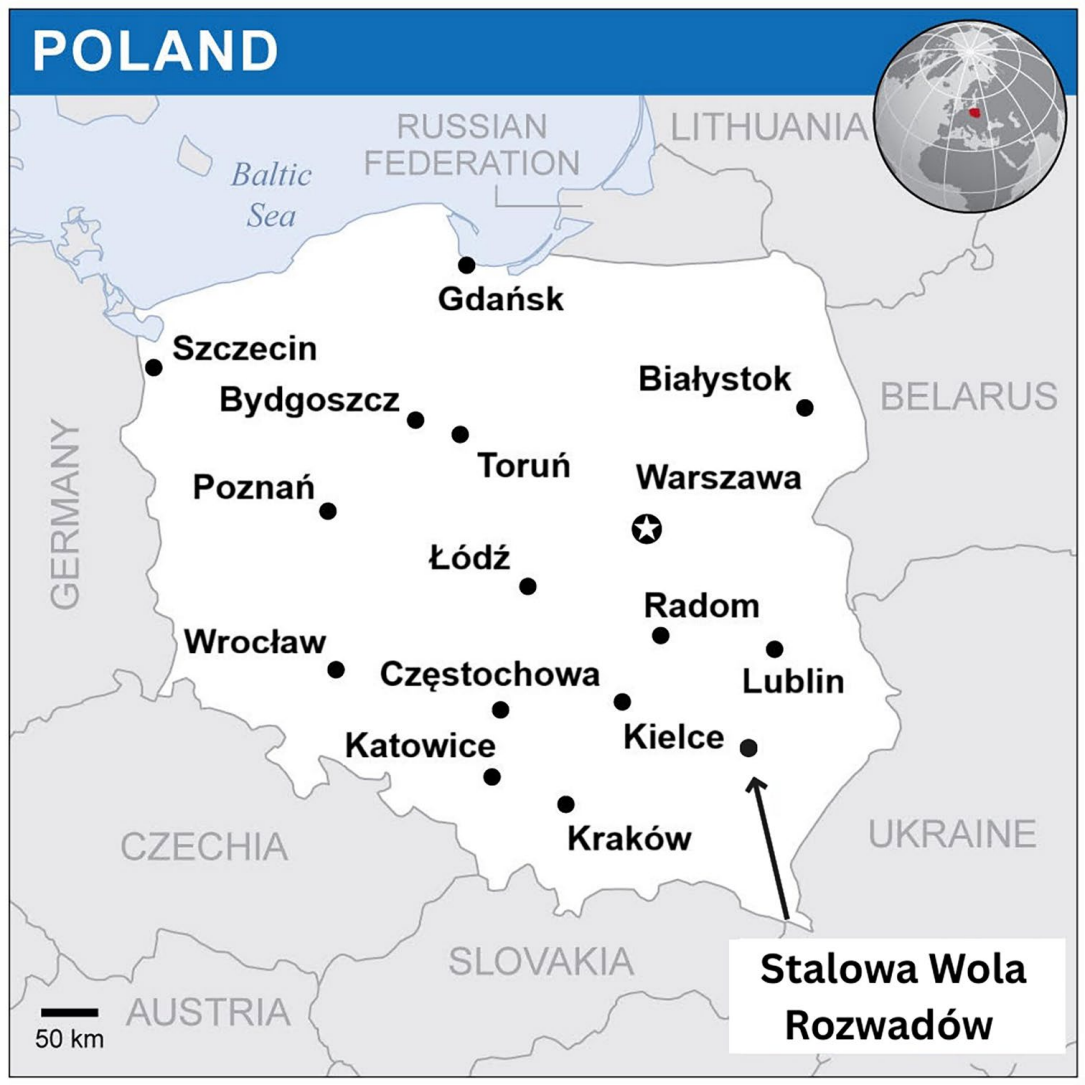
Map of Poland indicating Rozwadów. Source: UN Office for the Coordination of Humanitarian Affairs (OCHA), modified by the authors; https://reliefweb.int/map/poland/poland-location-map-2019

**Fig. 2:**
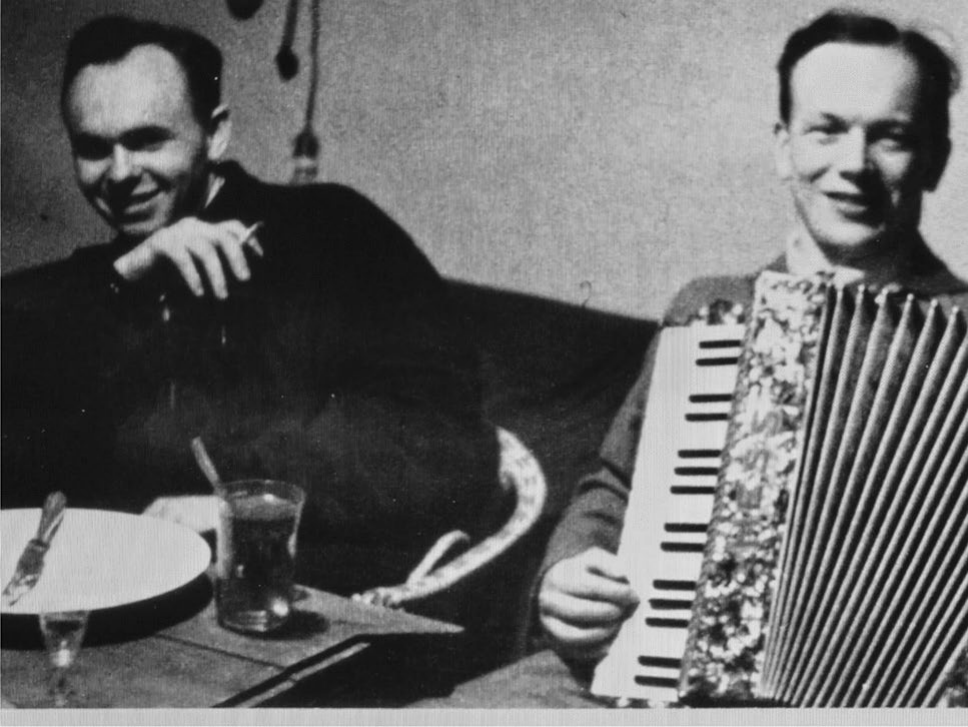
Photograph showing Eugeniusz Łazowski (with accordion) and Stanisław Matulewicz. Provided by Mark Gerard, grandson of Eugeniusz Łazowski

In 1991, the Chicago-based Polish paediatrician Eugeniusz Łazowski published his memoir *Private War—Memoirs of a Doctor Soldier 1933–1944*. In 2000, it was issued in Polish under the title *Prywatna wojna—Wspomnienia lekarza-żołnierza 1933–1944*. He recalled his life as a general practitioner in Rozwadów, a small town in southern Poland under German occupation during World War II, now part of Stalowa Wola [1, 2] (Fig. 1). The book describes a fabricated typhus epidemic, staged together with his colleague Stanisław Matulewicz (Fig. 2). The two physicians had already reported this episode in 1977 in *ASM News*, the monthly bulletin of the American Society for Microbiology [3]. That same year, the case was cited in the *British Medical Journal* [4] and later reprinted in *U.S. Navy Medicine* [5]. Although rarely cited in medical literature, the story attracted wider public attention through a *Chicago Tribune* article in 2006 [6] and the Polish-French documentary *Looking for a Polish Schindler* in 2019.

The method used to fabricate the epidemic was unique and has not been described elsewhere. The aim of this article is to review available evidence that may substantiate the reported events, place them in the context of existing medical literature, and present findings from archival documents in the city of Stalowa Wola.

### Epidemic typhus

Epidemic typhus is a severe illness with a mortality rate of about 4% when treated with antibiotics such as tetracycline or chloramphenicol, but reaching 10–60% without therapy in the pre-antibiotic era [7, 8]. It is caused by *Rickettsia prowazekii*, an obligate intracellular bacterium transmitted by the body louse *Pediculus humanus corporis*, which thrives in clothing at around 20 °C, and is named after Howard T. Ricketts and Stanislaus von Prowazek, who first described the organism and who both tragically succumbed to the disease during their research. Lice feeding on the blood of an infected host excrete rickettsiae in their faeces within three days; the bacteria can remain viable for up to 100 days and enter the human body through breaks in the skin or mucous membranes. The incubation period of the disease is 10 to 14 days. Endothelial cells of small capillaries are the main target of rickettsiae leading to vasculitis and multiorgan dysfunction. Symptoms include high fever, headache, severe myalgia, rash, and neurological manifestations such as delirium, seizures, or coma. Untreated disease lasts about two weeks, with recovery often taking months. Patients may remain latently infected for decades and later develop Brill-Zinsser disease, a milder relapse with mortality below 1% [9–11]. Such patients can act as reservoirs despite acquired immunity [9, 12]. Epidemics occurred most often in overcrowded, unhygienic conditions. Historically, epidemic typhus has caused millions of deaths, particularly during warfare. In Napoleon’s campaign against Russia, it is estimated that one-third of French soldiers died of typhus [13]. Charles Nicolle demonstrated that the body louse was the vector of typhus, a discovery that earned him the Nobel Prize in 1928. The discovery of louse transmission [14] and identification of *R. prowazekii* as the causative agent [7] enabled preventive measures, such as disinfection of clothing and lice eradication campaigns, first effectively applied during World War I. Nonetheless, mortality remained high in Eastern Europe, where overcrowding and shortages of disinfectants fuelled epidemics. During the Russian civil war after 1917, an estimated three million people died [15]. In newly independent Poland, large outbreaks occurred in 1918–1919, linked to the return of prisoners of war and refugees from the Soviet Union. Intensive national campaigns against lice and improved sanitation quickly reduced transmission, and by October 1920 the epidemic was contained [16].

In the 1930s, the Polish scientist Rudolf Weigl developed the most effective vaccine, using rickettsial cells harvested from the gastrointestinal tract of infected lice and inactivated with phenol. With an efficacy of about 80%, it became the cornerstone of typhus prevention [16–19]. During the interwar years, typhus was no longer regarded as a major threat in most countries.

This perception changed with the outbreak of World War II. German authorities feared the spread of infections from regions where typhus remained endemic, especially since most Germans lacked immunity and no effective vaccine was widely available to them. In the occupied Polish territories, organised as the “General Government” (GG), strict isolation of suspected cases became the main preventive measure. In a racist and antisemitic manner, typhus was officially portrayed as a disease spread particularly by Jews [16, 20], and affected Jewish persons were subject to immediate execution. German health authorities in the GG did not treat typhus as an ordinary infectious disease but framed it as a geopolitical and racially defined danger, to be addressed not by treatment but by eliminating those labelled as potential carriers [21]. This rationale underpinned forced relocation of Jewish populations into ghettos, where overcrowding, malnutrition, and poor hygiene fostered devastating outbreaks, as seen in the Warsaw ghetto [22]. It is estimated that more than 500,000 people died of typhus in ghettos across occupied Poland [23]. Outbreaks of typhus were also common in concentration camps, where many prisoners succumbed to this disease [24] or were executed after being diagnosed with it [21].

### Weil–Felix test

The standard diagnostic method for epidemic typhus during World War II was the Weil–Felix test, first described in 1916 [25]. The test is based on a structural and serological similarity between the O antigens of *Proteus vulgaris* OX19 (serogroup O1) and lipopolysaccharides of *R. prowazekii* [26]. Antibodies produced in patients with epidemic typhus cross-react with *P. vulgaris* OX19 cells, producing visible agglutination [27]. Although the immunological basis of this phenomenon was not understood at the time, the test was widely applied during the war.

Today, more sensitive and specific methods are available, such as polymerase chain reaction (PCR), indirect immunofluorescence assays, and enzyme-linked immunosorbent assays (ELISA) [11]. Nevertheless, the Weil–Felix test is still used in resource-limited settings because it is inexpensive and technically simple [11, 27, 28].

The assay can be performed in qualitative, semi-quantitative, or quantitative formats and is relatively easy to carry out. In the quantitative method, 0.1 mL of patient serum, obtained by centrifugation of naturally clotted blood, is serially twofold diluted in tubes containing 0.85% NaCl solution (1:20 to 1:1,280; final volume 1 mL). A drop (approximately 40–50 µL) of the appropriate Weil–Felix reagent—a suspension of *P. vulgaris* OX19 cells containing about 10^10 killed bacteria per mL [29] (THERMO)—is added to each tube. The mixtures are incubated for 24 h at 37 °C or for 4 h at 50 °C, and then examined for the presence of white granular agglutinates [27, 30].

Diagnostic thresholds have varied historically. At present, titres of 1:160 to 1:320 are considered positive, whereas during the time of Łazowski and Matulewicz, thresholds of 1:80 to 1:100 were already accepted [3, 5, 31]. Other reports indicate that titres as low as 1:40 could be regarded as diagnostic [32]. Currently, the Weil–Felix test is recognised as having low specificity and sensitivity. A century ago, however, its sensitivity was estimated at 90% and it was considered the gold standard in typhus diagnostics [32, 33]. By contrast, the manufacturer of a modern commercial reagent reports a sensitivity of about 70% [34].

Test performance also depends on the stage of infection. In the first week, agglutinin levels may be too low for detection, leading to false-negative results, whereas by the second week titres are more reliably detected. Antibody titres then gradually decline over subsequent weeks [3, 5, 27, 35]. Fairley [32] (1919) demonstrated this pattern, observing titres rise from 1:40 to as high as 1:640–1:1280 between the 5th and 13th day of illness, followed by a gradual decline during convalescence.

### The “typhus epidemic” in Poland from 1941 to 1944

Here we briefly summarise the description of the “epidemic” according to Łazowski [1]. During World War II, he worked as a general practitioner in Rozwadów. His colleague, Dr. Stanisław Matulewicz, had his practice nearby in Zbydniów, where he also operated a small improvised laboratory that enabled him to perform the Weil–Felix test.

In this laboratory, Matulewicz experimented with the Weil–Felix reagent, a suspension of phenol-killed *P. vulgaris* OX19 bacteria. In 1941, he applied it as an intramuscular injection to a Polish labourer who had been deported to Germany but was granted leave to visit his family in Rozwadów and asked Matulewicz to do anything possible, even mutilate him, to avoid returning as a forced worker [1]. Six days after the injection, without any noted adverse reaction, the man’s serum produced a positive Weil–Felix reaction (titre 1:500) [1, 3]. This “vaccination” demonstrated that a false diagnosis of typhus could be produced and gave Łazowski the idea of protecting local people from deportation by falsely diagnosing them as typhus patients.

According to this plan, the doctors would inoculate subjects who were febrile from other causes, with the *P. vulgaris* OX19 reagent in order to produce a positive Weil–Felix result. They explained the injections to their patients as “protein stimulation therapy,” a method widely used before the war that involved vaccines, autohemotherapy, or pharmaceutical preparations and was believed to stimulate the immune system [1, 3]. Blood samples from these patients were then sent to German laboratories, and when a positive result was confirmed, the “patients” were registered as typhus cases. According to Łazowski´s report this protected them from deportation to forced labour and from other atrocities of the occupiers. As the number of positive cases increased, the German authorities interpreted the results as evidence of an epidemic, and the region soon came to be regarded as an infectious hotspot and was consequently avoided.

Łazowski and Matulewicz soon realised that their plan worked as expected. They carefully imitated the natural course of an epidemic by increasing case numbers in the cold season and reducing them in spring and summer. In this way, many individuals were protected, although Łazowski did not provide exact numbers. He only stated that about a dozen villages in the area were declared “epidemic” [1, 3]. The danger of the endeavour was extreme, since exposure would have meant execution for both physicians, their families, and anyone else involved. For this reason, the action remained a strict secret, not even shared with their wives.

In 1944, suspicion arose among the German authorities, and a special commission was dispatched to investigate. Because of fear of infection, the investigators avoided direct examination of the “patients” and limited themselves to taking blood samples for testing. The Weil–Felix reactions were positive, and suspicions against Łazowski were lifted. In spring 1944, the number of reported cases was reduced as planned, and in July Łazowski fled with his wife and daughter to Stalowa Wola after falling under suspicion of helping the underground army. This suspicion was indeed justified, as he was involved in supporting the underground [1], and it was the decisive reason for his immediate decision to leave.

### Reception of the fabricated epidemic

After the first article by Łazowski and Matulewicz appeared in 1977, only a few papers in the medical literature commented on the event. That same year, the *British Medical Journal* published a brief summary [4]. In 1990, Bennett and Tyszczuk discussed the case from the perspective of military physicians, also highlighting the contributions of Rudolf Weigl to typhus vaccine development [36]. Despite four Nobel Prize nominations, he never received the award [36]. The story of Łazowski and Matulewicz was recounted by science writer Rebecca J. Anderson in [37]. More recently, Berger [38] underlined the ethical dimension and heroism of Łazowski’s and Matulewicz’s actions, interpreting them as a form of “self-sacrifice” comparable to the dedication of physicians during the COVID-19 pandemic. In 2022, a group of Polish dermatologists revisited the mock epidemic in connection with the work of Weigl and Ludwik Fleck, another key typhus researcher [39]. Finally, in 2023, Shanks discussed the episode in the context of other historical examples where infections or vaccines were used with the intention of immunological warfare [40]. None of these papers, however, attempted to verify the historical account with external evidence.

In contrast, the story has circulated widely in the public sphere. The *Chicago Tribune* reported on it in 1977 [41] and again in 2006 [6]. Several headlines suggested that Łazowski had saved the lives of 8,000 Jews, although this claim appears nowhere in his memoirs or articles. On the contrary, Łazowski noted that a Jew with typhus would have been executed immediately by the occupiers as a source of the “Jewish illness” [1, 20]. The myth that many Jews were saved was definitively refuted by Barbara Necek’s 2019 documentary *Looking for a Polish Schindler*, which showed that so many Jewish people had never lived in Rozwadów and that by 1941, when the two doctors began their action, most Jews in the region had already been murdered or deported to concentration camps. Nevertheless, Łazowski and his wife witnessed the execution of Jewish inhabitants on the Rozwadów market as late as July 1942 [1].

Cultural reception also emerged. In the novel *Night Train*, written by Barbara Wood and physician Gareth Wootton and first published in 1979, a fabricated typhus epidemic in wartime Poland plays a central role. The organisation of the “epidemic” is described in detail and closely mirrors Łazowski’s later account. According to his family, Łazowski was not involved in the writing of the novel, suggesting that the 1977 *ASM News* article and the contemporary *Chicago Tribune* coverage most likely served as the basis for its depiction.

### A critical assessment of the “epidemic”

The use of inactivated *Proteus* bacteria by intramuscular injection has not been described in the medical literature before or after the fabricated “epidemic” reported by Łazowski and Matulewicz. According to Łazowski, Matulewicz ran a basic laboratory on his estate, where he could perform the Weil–Felix test despite lacking specialised equipment. Thermoregulation at around 38 °C was achieved using an electric heater and a room thermometer, a simple but effective method of temperature control. The test could also be performed at room temperature, although results would appear more slowly [33]. With these simple means Matulewicz successfully analysed the sera of patients with clinically diagnosed typhus and provided titres. Rapid testing was particularly valuable when confirming the disease in individuals who needed to remain hidden from German authorities. He also observed that intramuscular injection of 1 mL of *P. vulgaris* OX19 (Weil–Felix reagent) suspension into a person without typhus produced a positive Weil–Felix reaction six days later, with a titre of 1:500 [1, 3, 5].

We are aware of only one documented case of intentional inoculation of humans with *Proteus* bacteria to test the Weil–Felix reaction. Fairley [32] (1919) reported subcutaneous inoculation of two laboratory staff members with a live bacterial suspension (then known as *Bacillus proteus*). Both developed a general systemic reaction lasting less than 48 h, and in one case a local reaction required surgical treatment. In both cases, agglutination occurred in the Weil–Felix test, with titres of 1:360 and 1:160 on the sixth day after inoculation. Effective immune responses have also been noted after intramuscular injection of inactivated cells of other Gram-negative bacteria, such as whole-cell vaccines against *Haemophilus influenzae* and *Bordetella pertussis* [42], or *R. prowazekii* in Weigl’s typhus vaccine [19]. At present, whole-cell vaccines containing killed *Proteus* strains and other uropathogens are administered orally to prevent urinary tract infections [43]. Earlier Russian studies examined subcutaneous injections of soluble antigens derived from *Proteus* cells, reporting both immunogenic and protective effects [44].

Animal studies lend further support to Łazowski’s observations. Intravenous application of inactivated *Proteus* suspension in rabbits elicited strong immune responses, with antisera reacting intensely against homologous strains. Agglutination titres reached 1:5,120, corresponding to ELISA titres of 1:512,000 [45–48]. Similarly, soluble-antigen vaccines were immunogenic in mice, guinea pigs, and rabbits [44], while subunit intranasal vaccines using *P. mirabilis* fimbrial proteins produced strong and protective immune responses in murine models [43, 49].

The antibody response to homologous *P. vulgaris* antigens in vaccinated animals was stronger than that of *R. prowazekii* antibodies in human infection. Two studies from the 1930s [50] and from 1998 [48] showed that sera from rabbits immunised with a suspension of *P. vulgaris* OX19 cells reacted with *P. vulgaris* OX19 cells/LPS much more strongly than sera from people suffering from typhus. Additionally, Dammin et al. [51] showed that *Proteus* OX19 infections alone can cause a positive Weil–Felix result because of the shared antigens between these two organisms. Taken together, these findings suggest that inoculation with *Proteus* is sufficient to produce a positive Weil–Felix result within six days, consistent with the report of Łazowski and Matulewicz.

Łazowski and Matulewicz purchased suspensions of phenol-killed *P. vulgaris* OX19—the reagent for both testing and inoculation—from a Warsaw laboratory supply shop, “Dom Medycyny” [2] (House of Medicine). Produced by the National Institute of Hygiene, it was easily accessible [1–3, 5]. Contemporary sources describe the Weil–Felix test as the gold standard for typhus diagnosis, and laboratories were expected to stock the reagent routinely [31]. Assuming reagent volumes similar to today’s 5 mL bottles [29], one vial would have sufficed to inoculate five people. The bacteria were inactivated by phenol or formalin, eliminating infection risk. Adverse reactions should be rare and mild, comparable to those seen with other inactivated bacterial vaccines, which are reactogenic but seldom cause serious complications [52].

Łazowski also treated patients with real typhus, giving him experience in managing the disease. The occurrence of sporadic genuine cases was a necessary precondition for the doctors’ plan; without them, the sudden emergence of a new epidemic would likely have prompted German investigation. Such an inquiry occurred only in the final stage of the epidemic in 1944, when suspicions arose that Łazowski might have been relabelling blood samples from true typhus patients. To counter this, German doctors themselves drew blood from “sick” individuals—actually inoculated with *Proteus*—and all samples tested positive in their laboratory [1, 3, 5]. One might wonder why no false-negative results were reported. A possible explanation is that Łazowski carefully selected Weil–Felix positive patients likely to yield consistent results when examined by the committee. Reports of the Weil–Felix test’s sensitivity vary widely (30–90%). Empirical data suggest that antibody titres against *P. vulgaris* OX19 are consistently higher after *Proteus* immunisation in animals than after infection in humans with *R. prowazekii* [48]. Thus, it seems highly plausible that the commission found no patient with a negative Weil–Felix reaction.

Łazowski and Matulewicz also demonstrated a sophisticated understanding of epidemiology. They increased the number of inoculations in winter and reduced them in summer, modelling the seasonal pattern of typhus epidemics. This further convinced the German authorities of the epidemic’s authenticity.

We are not aware of German laboratory reports confirming the epidemic, since most documents were destroyed by the occupiers before their retreat. Our own inquiries in Stalowa Wola (Rozwadów) were fruitless, as the local archive was destroyed after the war. However, we identified an independent document describing a fake typhus epidemic in the region: the memoirs of Father Albin Blajer, typewritten in 1974 and held in the parish library of Grębów, Poland. Blajer, a priest active in the underground and in secret teaching, reported numerous fake “typhus” deaths in the nearby village of Turbia, which protected people from deportation to labour camps. To create the appearance of an epidemic, he compiled lists of “typhus” victims by including individuals who had in fact died of other causes. According to Blajer, people in the area were aware of the fabrication, and we may conclude that they knew the actual deaths in their village were not mainly due to typhus. He also described a commission that investigated the cases but found no irregularities, confirming the same event described by Łazowski. The priest added that the church was closed for four months during winter as a supposed prophylactic measure against mass gatherings, and people were also spared from being sent to forced labour, while food contingents were not confiscated.

The names of Łazowski and Matulewicz are not mentioned in this document, as they likely never knew of each other. Blajer recalled submitting his lists to a district doctor named Rzucidło, who also appears in Łazowski’s memoirs as the person who warned him about the commission. Written before Łazowski and Matulewicz’s 1977 article, Blajer’s testimony is considered independent, and Łazowski’s family is not aware of any meeting between them (Mark Gerrard, personal communication). This supports the authenticity of Blajer’s report as an independent document, supporting the reality of a fabricated epidemic. In contrast to Łazowski’s account, Blajer stated that his fabrication was widely known in the local population. However, people routinely concealed information from German occupiers, especially when disclosure could endanger them. In this context, silence was not only protective but essential: any form of conspiratorial activity was punishable by death, making strict secrecy indispensable. Taken together, the convergence of both accounts provides convincing evidence that the false typhus epidemic truly occurred. Though uncoordinated, the actions of Father Blajer and the two physicians complemented each other—his administrative fabrication reinforced their immunological “private war”.

## Conclusion

The fake epidemic of typhus reported by Łazowski and Matulewicz is unique in history and has not been corroborated by other sources. Our review of the available evidence revealed no inconsistencies that would call it into question. An independent account by the contemporary priest Albin Blajer does not reproduce Łazowski’s narrative, but it confirms the occurrence of a fabricated typhus epidemic at that time. It also underlines similar effects on the population, as described by Łazowski, while not contradicting his report. Taken together, these findings support the credibility of the story and its place as an exceptional episode in medical and wartime history.

It is remarkable that this event has attracted so little attention in the medical literature. Several aspects deserve special recognition. Notably, the idea to inoculate people with *P. vulgaris* bacteria in order to obtain a positive reaction in the Weil–Felix test was highly original. Under normal circumstances such a procedure would have had no benefit for the recipients. Under the conditions of German occupation, however, where typhus was regarded as a dangerous and untreatable infection, a positive test result paradoxically became protective, although the risk of execution remained and protection was never assured. Combining this laboratory observation with the historical context was the key insight of Łazowski and Matulewicz. Their intervention is thus unique in that it was not aimed at preventing a disease, but at preventing social persecution.

The actions of the two physicians testify to both their humanity and their sense of responsibility as members of an oppressed population. It also required considerable courage to deceive the German authorities, since discovery would have meant immediate execution. After the war, when the danger had passed, they did not promote themselves as heroes, and their story was nearly forgotten. It is therefore timely to recall this extraordinary episode and to recognise Eugeniusz Łazowski and Stanisław Matulewicz as exemplary figures whose ingenuity and moral commitment under an oppressive system remain relevant for physicians today.

## Conflict of interest

The authors declare no competing interests.

## Data Availability

No datasets were generated or analysed during the current study.
